# NucAmino: a nucleotide to amino acid alignment optimized for virus gene sequences

**DOI:** 10.1186/s12859-017-1555-6

**Published:** 2017-03-01

**Authors:** Philip L. Tzou, Xiaoqiu Huang, Robert W. Shafer

**Affiliations:** 1 Division of Infectious Diseases, Department of Medicine, Stanford University, Stanford, CA USA; 2 Iowa State University, Ames, IA USA

**Keywords:** Sequence alignment, Viruses, HIV-1, Drug resistance, Open source

## Abstract

**Background:**

Current nucleotide-to-amino acid alignment software programs were developed primarily for detecting gene exons within eukaryotic genomes and were therefore optimized for speed across long genetic sequences. We developed a nucleotide-to-amino acid alignment program NucAmino optimized for virus sequencing.

**Results:**

NucAmino is an open source program written in the high-level language Go. NucAmino is more likely to align codons flush with a reference sequence’s amino acids and can be modified to facilitate the placement of insertions and deletions at specific positions. We compared NucAmino to the nucleotide to amino acid alignment program Local Alignment Program (LAP) using 115,118 human immunodeficiency virus type 1 (HIV-1) protease, reverse transcriptase, and integrase sequences—three genes that are commonly sequenced in clinical laboratories. Discordances between NucAmino and LAP occurred in 512 (16.9%) of the 3,029 sequences containing gaps but in none of 112,910 sequences without gaps. For 242 of the sequences with discordances, NucAmino produced an alignment that was preferable to that found by LAP in that it was more likely to codon align insertions and deletions and to facilitate the placement of an important drug-resistance associated insertion at the position at which most laboratories expect it to occur.

**Conclusions:**

NucAmino is a nucleotide-to-amino acid alignment program with several advantages for clinical laboratories performing virus sequencing compared with older programs designed for gene finding.

## Background

The molecular targets of human immunodeficiency virus type 1 (HIV-1) therapy including reverse transcriptase (RT), protease, and integrase are among the most commonly sequenced genes in clinical laboratories. In many countries, these genes are sequenced routinely in patients before starting HIV-1 drug therapy and in patients with virological failure while on therapy. Such sequencing is usually performed using direct polymerase chain reaction (PCR) dideoxy-nucleotide Sanger sequencing. Nucleotide sequences are then aligned to a reference amino acid sequence to identify amino acid substitutions, insertions, and deletions associated with reduced drug susceptibility.

Current nucleotide to amino acid alignment programs such as Local Alignment Program (LAP) [1] and GeneWise [2] were developed primarily for detecting gene exons within eukaryotic genomes and were therefore optimized for speed across long genetic sequences and for handling intron-exon boundaries. We sought to develop a nucleotide-to-amino acid alignment program optimized for virus sequencing rather than gene finding. As such our program was designed to handle IUPAC ambiguity codons caused by electrophoretic mixtures reflecting the multiple virus variants usually present within a sequenced sample and to ignore the possibility that a codon may be disrupted by an intron.

More importantly, virus genotyping may require certain insertions or deletions to be placed at a specific position even if an optimized alignment may place the insertion or deletion at a different position. For example, insertions in the HIV-1 RT β3-β4 loop, encompassing residues 64 to 72, are traditionally placed at residue 69 because most genotypic resistance interpretation software associates this insertion (but not others at nearby positions) with high-level resistance to several nucleoside RT inhibitors [3, 4]. This problem arises because, in highly variable viruses, nucleotide and amino acid variability surrounding indels often influences indel placement during pairwise sequence alignment.

Here we describe a program called NucAmino designed to align a virus nucleotide sequence to a reference amino acid sequence. We compare the performance of this program to LAP for the analysis of HIV-1 sequences from 115,118 individuals. We also used these sequences to compare NucAmino with JAligner—a commonly used implementation of the Smith-Waterman algorithm for aligning two nucleotide sequences [5, 6]. We show that NucAmino is likely to be useful for clinical laboratories performing HIV-1 genotypic resistance testing that must handle polymorphic virus sequences containing electrophoretic mixtures and requiring consistent placement of certain indels.

## Implementation

**Fig. 1:**
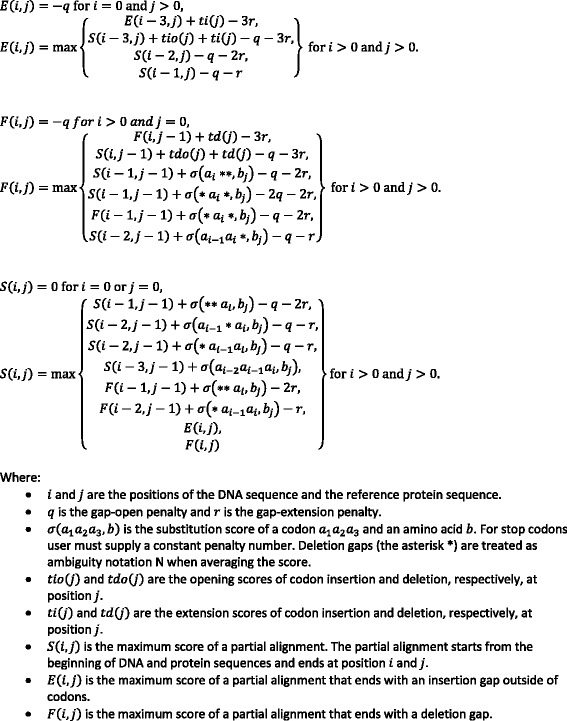
Dynamic programming alignment implemented by NucAmino to align nucleotide sequences to a reference amino acid sequence

The dynamic programming algorithm used by NucAmino performs an optimal local alignment of a nucleotide sequence to a reference amino acid sequence using an amino acid substitution matrix, partial scores for codons containing ambiguous IUPAC nucleotides, and a positional scoring matrix to facilitate the consistent placement of certain indels. The algorithm is presented in Fig. 1. NucAmino is written in the computer language Go and can be retrieved from Github (https://github.com/hivdb/NucAmino). Users can download the executable files for Linux, Mac, or Windows or build NucAmino following the instructions in a Readme file.

The NucAmino algorithm has six differences from the dynamic programming algorithm used by LAP. Three are simplifications reflecting the fact that NucAmino does not attempt to identify exons separated by introns: (i) NucAmino does not align the reverse complement of a nucleotide sequence to the reference amino acid sequence; (ii) NucAmino does not consider the possibility that a gap may exist inside of a codon—as when an intron interrupts a codon; and (iii) NucAmino uses penalties for gap opening and gap extension but not for capping the gap extension penalty.

Three of the six differences between NucAmino and LAP are features developed specifically for the alignment of virus sequences: (i) To facilitate codon alignment (the alignment of indels flush with amino acid positions), NucAmino accepts user-defined constant optional opening and extension bonus scores for gaps that are multiples of three that begin and end at a codon boundary; (ii) To facilitate the precise specification of the position of an insertion or deletion, NucAmino accepts a positional indel matrix containing a list of scores for indels at particular positions. For example, in our implementation, the positional indel matrix has the following entry “RT, insertion, 69, +6”; and (iii) NucAmino translates codons containing an IUPAC ambiguity to one or more amino acids and then assigns the score for the alignment of that codon to the reference amino acid by averaging the BLOSUM62 scores associated with each translated codon.

To compare NucAmino and LAP, we used plasma virus sequences determined by direct PCR dideoxy-nucleotide sequencing of HIV-1 protease, RT, and/or integrase complementary DNA (cDNA) from 115,118 individuals in the Stanford HIV Drug Resistance Database [7]. The complete set of sequences and their GenBank accession numbers are available in the NucAmino Github repository. For this comparison, both NucAmino and LAP aligned each sequence to the 948 amino acid subtype B consensus HIV-1 pol amino acid sequence comprising protease, RT, and integrase (https://www.hiv.lanl.gov/content/sequence/HIV/CONSENSUS/Consensus.html) using a gap-opening penalty of 10 and a gap-extension penalty of 2. Both also used the BLOSUM62 substitution matrix.

NucAmino also assigned opening and extension bonus scores of 0 and 2, respectively, for codon-aligned indels. The indel positional matrix had a bonus score of 6 for RT codon 69. There have been two other well-described regions with indels in these HIV-1 genes: deletions in the RT β3-β4 loop region [4, 8] and insertions between codons 33 and 41 in protease [9]. NucAmino did not include scores for these in the indel positional matrix, because each of the RT β3-β4 loop deletions are associated with different drug-resistance interpretations and because the protease codon 33/41 insertions are not associated with drug resistance.

NucAmino and LAP results for each sequence included (i) a list of the genes in the sequence (protease, RT, and/or integrase), (ii) the gene boundaries according to the reference amino acid sequence (first amino acid and last amino acid), (iii) a list of amino acid differences from the reference which we refer to as mutations, and (iv) a list of gaps. Gaps included insertions, deletions, and frameshifts. Gaps that were multiples of three nucleotides and aligned flush to one or more codons were classified as insertions or deletions (i.e., indels). Gaps that were just one or two bases were called frameshifts.

To compare NucAmino with JAligner, we used JAligner to align each of the sequences described above to the consensus subtype B nucleotide sequence. We used this secondary analysis to determine whether nucleotide-to-amino acid alignment had an advantage over a nucleotide-to-nucleotide alignment for the optimization of gap placement.

## Results and Discussion

Of the 115,118 HIV-1 sequences, 61.2% had protease and RT, 15.9% had just protease, 14.4% had just RT, 5.0% had just integrase, and 3.5% had protease, RT, and integrase. Overall, the sequences averaged 1,178 nucleotides in length. Nucleotide-to-amino acid alignments produced by NucAmino and LAP were completely concordant in identifying the same amino acid mutations, indels, and frameshifts for 99.6% of sequences. However, discordances between NucAmino and LAP occurred in 512 sequences—0.4% of the total and 16.9% of the 3,029 sequences containing gaps—but in none of the sequences without gaps. NucAmino and LAP identified a mean of 20.8 and 20.9 amino acid mutations per sequence, respectively.

NucAmino and LAP identified insertions in 475 (0.41%) sequences. The most commonly occurring insertions were in the β3-β4 loop region of RT between codons 62 and 72 and between codons 33 and 41 of protease. Of 232 insertions in the RT β3-β4 loop region, 195 (84.1%) were double amino acid insertions, 30 (12.9%) were single amino acid insertions, and 7 (3.0%) were insertions with more than two amino acids. NucAmino codon-aligned each of these insertions. In contrast, LAP codon-aligned 153 (65.9%) of these insertions; whereas 79 (34.1%) were not aligned flush with reference amino acid positions.

NucAmino, through the use of its positional insertion matrix, placed all but three of the insertions at codon 69. In contrast, LAP placed 113 insertions (48.7% of 232) at codon 69. It placed 111 insertions (47.9% of 232) at codons 63 (n = 1), 64 (n = 2), 65 (n = 5), 66 (n = 51), 67 (n = 2), 68 (n = 45), and 70 (n = 5). The remaining 8 insertions (3.4% of 232) were not appropriately codon aligned. The three insertions that were placed by LAP at codons 63 and 64 were placed at codons 62 by NucAmino. Overall, differences in insertion placement in this region were responsible for 125 of the 512 discordances between NucAmino and LAP (Table 1).

**Table 1:** Classification of Sequences with Gaps for Which Local Alignment Program (LAP) and NucAmino Yielded Different Results

Classification	Number of Discordances	Explanation	Example	
			LAP output	NucAmino output
A. Insertions and deletions in RT β3–β4 loop region (codons 62 to 72)	128	For 233 insertions in this region, NucAmino placed all but 3 at codon 69. In contrast, LAP placed 113 insertions at codon 69, whereas 111 were placed at codons 62 to 70. For 99 deletions in this region, there were 3 differences between NucAmino and LAP which can be all classified into classification D. (Example shown on the right: AF315241)	66 67 68 69 70 Lys Asp Ser Thr Lys : : : . . . ++++++ : : : . . . : : : AAA GAA AGTTCT AGC GGT AAA	66 67 68 69 70 Lys Asp Ser Thr Lys ::: ... ::: . . . ++++++ ::: AAA GAA AGT TCT AGCGGT AAA
B. Insertions in PR codons 33/41 loop region	31	For 151 insertions in this region, LAP and NucAmino placed 31 insertions at different positions. (Example shown on the right: HQ657812)	32 33 34 35 36 37 Val Leu Glu Glu Met Asn ::: ::: +++ ::: ::: ... ::: GTA TTA GAR GAA GAA ATA AAT	32 33 34 35 36 37 Val Leu Glu Glu Met Asn ::: ::: ::: ::: +++ ... ::: GTA TTA GAR GAA GAA ATA AAT
C. Different placement of indels and/or frameshifts (not in classification A or B)	213	For 213 sequences with indels and/or frameshifts outside of the RT β3–β4 loop region and the PR codon 33/41 loop region, gaps were placed at slightly different positions. (Example shown on the right: EF071939)	306 307 308 309 310 311 312 Asn Arg Glu Ile Leu Lys Glu ::: ... ... --- ... ::: ::: AAC AAG AAT TTT AAG GAG	306 307 308 309 310 311 312 Asn Arg Glu Ile Leu Lys Glu ::: ... ... ... --- ::: ::: AAC AAG AAT TTT AAG GAG
D. Codon alignment corrections (not in classification A, B or C)	108	Overall, there were 218 sequences with 110 insertions and 133 deletions for which LAP aligned 3 nucleotides across more than one codon whereas NucAmino aligned the nucleotides to a single codon. (Example: HM569289) Of these, 114 were not in the RT β3β4 loop or in PR codon 35/41 loop region.	200 2 01 202 203 Ile V al Asp Ile ::: .+++.. ::: ::: ATA GGAATA GGC ATA	200 201 202 203 Ile Val Asp Ile ::: +++ ... ::: ::: ATA GGA ATA GAC ATA
E. Large gaps	32	32 sequences had large gaps presumably because the contributor excluded unsequenced regions from the GenBank submission or inserted large stretches of N’s. For 21 and 11 of these regions, LAP and NucAmino accurately reported a large deletion encompassing the missing region, respectively. (Examples in the online dataset: HQ685003, AY090840)	Insufficient space to provide an example.	

*Abbreviations*: *RT* reverse transcriptase, *PR* protease

Of the 151 protease codon 33/41 insertions, 119 (78.8%) were single amino acid insertions, 31 (20.5%) were double amino acid insertions, and 1 (0.7%) was a triple amino acid insertion. NucAmino codon-aligned each of these insertions. In contrast, LAP codon-aligned 127 (84.1%) of these insertions; whereas 24 (15.9%) were not aligned flush with reference amino acid positions. Both NucAmino and LAP most often placed the insertion at codon 35 (58% for LAP and 62% for NucAmino). As noted in the Implementation section, NucAmino did not include positive scores for insertions at any of the positions in this region. Differences in insertion placement in this region were responsible for 31 of the 512 discordances between NucAmino and LAP (Table 1).

NucAmino and LAP identified 399 sequences (0.35% of the total number analyzed) containing one or more deletions, respectively. The most common deletions were in the RT β3-β4 loop region. Of the 99 deletions in this region, all were single amino acid deletions. NucAmino codon aligned each of these deletions, whereas LAP codon-aligned all but three of the deletions. Both methods placed the deletions most often at position 69 (53 for both NucAmino and LAP) or position 67 (33 for both NucAmino and LAP). As noted in the Implementation section, NucAmino did not include positive scores for deletions at any of the positions in this region. Differences in deletion placement in this region were responsible for 3 of 512 discordances between NucAmino and LAP (Table 1).

The remaining discordances between NucAmino and LAP occurred in sequences with gaps outside of the RT β3-β4 and protease codon 33/41 loop regions. These occurred primarily in sequences from a small number of publications with missing nucleotides and probable sequencing or data entry errors. Most differences between NucAmino and LAP resulted from the placement of indels at slightly different positions. Several resulted from NucAmino’s greater likelihood of codon-aligning indels. Whereas, several resulted from LAP’s increased ability to tolerate large gaps (Table 1).

There were more discordances between NucAmino and JAligner than between NucAmino and LAP. JAligner incorrectly introduced gaps into 625 sequences that were not found to have gaps by NucAmino and LAP. JAligner detected 183,043 of the 183,453 (99.8%) DRMs detected by NucAmino and LAP. However, JAligner also detected 2,024 DRMs that were not detected by NucAmino or LAP. These DRMs uniformly resulted from differences in gap placement that resulted in incorrect reading frames. This comparison shows that nucleotide-to-nucleotide alignments can be a starting point for the alignment of a coding virus sequence to a nucleotide reference sequence but that additional post-processing using an amino acid reference sequence is required to avoid the placement of inappropriate gaps [10].

The algorithmic time complexity is *O*(*MN*)for both NucAmino and LAP. However, algorithmic space complexity is *O*(*MN*) for NucAmino and *O*(*N*) for LAP due to the latter program’s use of a linear space optimization that was first described by Myers and Miller [11]. The average processing time for aligning a sequence of ~1,200 nucleotides to the reference sequence of 948 amino acids was 45.2 ms for NucAmino and 30.1 ms for LAP on an iMac with 4 cores Intel Core i5 3.2 GHz and 32 GB of memory.

## Conclusions

NucAmino is a nucleotide to amino acid alignment program with several advantages compared with existing programs for clinical laboratories performing HIV-1 genotypic resistance testing. NucAmino can be modified to facilitate the placement of insertions and deletions at specific positions and is more likely to align codons flush with the reference sequence’s amino acids. Additional scoring adjustments are possible to fine tune alignments based on knowledge about the sequenced gene.

In contrast to LAP, NucAmino is open source and available for both academic and commercial laboratories. In contrast to LAP and GeneWise, which are written in C, NucAmino is written in the high-level language Go, making its code more comprehensible and modifiable. Although NucAmino is slower than LAP, speed is not an important factor for aligning individual standard dideoxy-nucleotide gene sequences. NucAmino would be considered slow for viral next-generation sequencing which typically yields about 1,000 to 10,000 reads per sample and would therefore typically require about one to ten minutes per sample on the iMac we used for testing. Ongoing optimizations to the Go compiler and the introduction of additional code to implement linear space optimization are possible future developments that would increase program speed as much as 10-fold [11, 12].

## Abbreviations

cDNA: Complementary DNA
HIV-1: Human Immunodeficiency Virus Type 1
IUPAC: International Union of Pure and Applied Chemistry
LAP: Local Alignment Program
PCR: Polymerase chain reaction
RT: Reverse transcriptase
